# LP-284, a small molecule acylfulvene, exerts potent antitumor activity in preclinical non-Hodgkin's lymphoma models and in cells deficient in DNA damage repair

**DOI:** 10.18632/oncotarget.28454

**Published:** 2023-06-12

**Authors:** Jianli Zhou, Drew Sturtevant, Cassie Love, Aditya Kulkarni, Neha Biyani, Umesh Kathad, Elizabeth Thacker, Sandeep Dave, Kishor Bhatia

**Affiliations:** ^1^Lantern Pharma Inc., Plano, TX 75024, USA; ^2^Department of Medicine, Duke University, Durham, NC 27708, USA; ^3^Data Driven Bioscience, Durham, NC 27707, USA

**Keywords:** non-Hodgkin's lymphoma, DNA damage, homologous recombination repair, transcription-coupled nucleotide excision repair, ATM

## Abstract

Despite advances in therapies treating non-Hodgkin’s lymphoma (NHL), 20~40% of patients experience relapsed or refractory disease. While solid tumors with homologous recombination deficiencies have been successfully targeted with synthetic lethal agents such as poly-ADP ribose polymerase (PARP) inhibitors, such synthetic lethality strategy has not yet been approved to treat patients with NHL. Here we investigated the mechanism of action (MoA) and therapeutic potential of a new-generation acylfulvene compound, LP-284, in both *in vitro* and *in vivo* NHL models. One of LP-284’s MoA includes inducing the repair of double-strand DNA break (DSB). We found that LP-284 exerts nanomolar potency in a panel of hematological cancer cell lines including fifteen NHL cell lines. *In vivo*, LP-284 treatment prolongs the survival of mantle cell lymphoma (MCL) cell line JeKo-1 derived xenograft mice by two-fold and shows increased efficacy over bortezomib and ibrutinib. In addition, LP-284 is capable of inhibiting tumor growth of JeKo-1 xenografts that are refractory to bortezomib or ibrutinib. We further showed that LP-284 is particularly lethal in cells with deficient DNA damage response and repair, a targetable vulnerability in NHL.

## INTRODUCTION

Non-Hodgkin’s lymphomas (NHL) are the seventh most prevalent class of cancers in the US. Mantle cell lymphoma (MCL) and diffuse large B cell lymphoma (DLBCL) are two major classes of NHLs and account for about 6% and 31% of all NHL cases, respectively [1]. Nearly all MCL patients relapse after standard frontline chemo-immunotherapies and have poor complete response rates of 19–71% after treatment with second-line therapies [2]. Patients with DLBCL have similar outcomes with approximately 30–40% of patients developing refractory or relapsed disease [3]. NHL patients typically experience poorer prognoses after each relapse, especially for those carrying unfavorable biomarkers such as TP53 [4] and dual translocation of MYC and BCL2/6 [5]. Therefore, new approaches that target the vulnerabilities in malignant NHL cells are always needed.

Alterations in DNA damage repair (DDR) represent a targetable vulnerability of many lymphomas [6]. Precisely targeting lymphomas that carry deficiency in the repair of DNA damage can be a strategy with clinical applications akin to the successes of synthetic lethal agents such as poly-ADP ribose polymerase (PARP) inhibitors. Molecular profiling of 2523 lymphoma samples across various histologic subtypes demonstrated that DDR is dysregulated in ~18% of the samples, with MCL (~47%), DLBCL (~21%), and chronic lymphocytic leukemia/small lymphocytic leukemia (CLL/SLL, ~15%) having the highest percentage of DDR dysregulation (Batlevi et al., 2022 American Society of Hematology conference abstract # 4169).

DDR is an intricate and sophisticated system where different types of DNA damage are repaired through different routes. Understanding the specific DNA damage and the genes involved in the corresponding repair pathway would help pinpoint targeted NHL patient populations. There are five major DDR pathways: homologous recombination repair (HR), non-homologous end joining (NHEJ), nucleotide excision repair (NER), base excision repair (BER), mismatch repair (MMR). The HR pathway provides a high-fidelity mechanism to repair DSBs, the most lethal form of DNA damage [7]. Due to the need for pairing with complementary sequences, HR repair typically occurs in S and G2 phases. The ataxia-telangiectasia mutated (ATM) protein plays a central role in the entire HR process through the phosphorylation of a battery of downstream HR factors such as H2A.X variant histone (H2AX) and breast cancer gene 1 (BRCA1). Cells carrying dysfunctional ATM have reduced HR activities and are reported to be hypersensitive to DNA-damaging agents [7, 8]. DSBs can also be repaired by the error-prone NHEJ pathway which mostly occurs in G1 phases [9].

The NER pathway corrects bulky base adducts introduced by ultraviolet light and certain chemical compounds such as cisplatin. NER can be divided into two sub-pathways: global genomic NER (GG-NER) and transcription-coupled NER (TC-NER). While GG-NER repairs DNA lesions throughout the genome, TC-NER only repairs damages that occurred on the transcribed strand of actively transcribing genes. TC-NER is an orchestrated process in which a group of ERCC proteins (ERCC1/2/3/4/5/6/8) play essential roles in damage recognition, DNA unwinding, and incision [10]. BER corrects small base lesions caused by oxidation, alkylation, methylation, or deamination [11]. MMR mainly fixes base-base mismatch, insertion, and deletion that occur during DNA replication and recombination [12].

At the cellular level, failure to repair damaged DNA, which typically occurs due to defective DDR or excessive DNA damage, or both, can lead to cell cycle arrest, apoptosis, or senescence. In fact, accumulated evidence has demonstrated the efficacy of DDR inhibitors as monotherapy or in combination in preclinical lymphoma models. A number of these DDR inhibitors have also been exploited in early clinical trials in lymphoma [6]. Mutation of ATM occurs in 40~50% of MCL patients [13] and ~14% of CLL patients [14]. Beyond mutations of genes directly involved in HR, deficiency in the HR pathway termed BRCAness has also been described for several hematological cancers [6]. The hallmark BCR-ABL fusion in chronic myeloid leukemia (CML) and high expression of LMO2 in DLBCL both lead to BRCAness or HR deficient (HRD) phenotype [6, 15]. Therefore, introducing DSBs that cannot be repaired efficiently in HRD lymphoma tumors creates a synthetic lethality treatment approach.

Alkylating agents have been tremendously successful in cancer therapy for decades due to their ability to damage DNA in fast-growing tumor cells and prevent cells from undergoing replication [16]. BER, MMR, and alkyltransferase are the major mechanisms for removing alkylated lesions. Unrepaired alkylated lesions can turn into DSBs after DNA replication, which subsequently makes HRD cells vulnerable to alkylating agents [17]. The R-CHOP regimen, consisting of rituximab and chemotherapies such as the alkylating agent cyclophosphamide, has remained the front-line therapy in NHL [18]. In addition to R-CHOP, bendamustine has been widely used in NHL treatment since its approval in 2008 [19].

Acylfulvenes are a class of alkylating agents with selective cytotoxicity toward cancer cells. One of the most extensively characterized acylfulvene compounds is irofulven, which generates DNA lesions that cause synthetic lethality in cancer cells with defective TC-NER, but not GG-NER [20, 21]. Another well-characterized compound LP-184 ((-)N-hydroxy-N-(methylacylfulvene)urea) has been shown to be selectively toxic in cancer cells with impaired TC-NER or HR (Kulkarni et al., 2022 American Association for Cancer Research conference abstract # B033). Comparing the cell cytotoxicity of acylfulvenes and conventional alkylating agents in the NCI-60 cell line panel has revealed a distinct antitumor spectrum of acylfulvenes [22], which is likely due to the different DDR pathways acylfulvenes invoke. On the one hand, cyclophosphamide and melphalan can induce O6-alkylguanine which can be repaired by alkyltransferase DNA repair. Bendamustine, a widely used alkylator for B-cell lymphoma, induces BER [23]. Cisplatin also induces TC-NER, but its resistance has also been linked to MMR deficiency [24]. On the other hand, acylfulvenes mainly alkylate adenine at position N3 or guanine at position N7 and their activities are independent of alkyltransferase repair, BER, or MMR [20]. Antitumor activities of acylfulvenes are also independent of TP53 status, increased expression of P-glycoprotein, or overexpressed multi-drug resistant protein-1, all of which contribute to resistance to conventional alkylating agents [25].

Both irofulven and LP-184 are pro-drugs that require activation by the NADPH-dependent oxidoreductase prostaglandin reductase 1 (PTGR1) [22]. The dependence on PTGR1 provides a patient-selection strategy for irofulven and LP-184 but limits their applications in cancer cells with low PTGR1. We sought to generate new acylfulvenes to extend the clinical scopes of irofulven and LP-184. The compound LP-284, or (+)N-hydroxy-N-(methylacylfulvene)urea, is the positive enantiomer of LP-184. Our previous studies demonstrate that LP-284 doesn’t require PTGR1 for activation, but retains strong antitumor activities in the NCI-60 human tumor cell line panel (Zhou et al., 2022 Society of Hematologic Oncology conference abstract # MCL319).

Here, we aimed to characterize LP-284’s antitumor efficacy in NHL models and further elucidate its mechanisms of action. In particular, we focused on MCL and double-hit lymphoma (DHL) where exist greater unmet medical needs as well as recognized deficiencies in DDR. We further investigated LP-284-induced damage at the DNA level and its selective lethality in DDR-deficient models, paving the rationale for clinical studies in NHL with DDR deficiency.

## RESULTS

### LP-284 is a new acylfulvene compound with robust anti-tumor activities in NHL cancer cell lines

**Figure 1:**
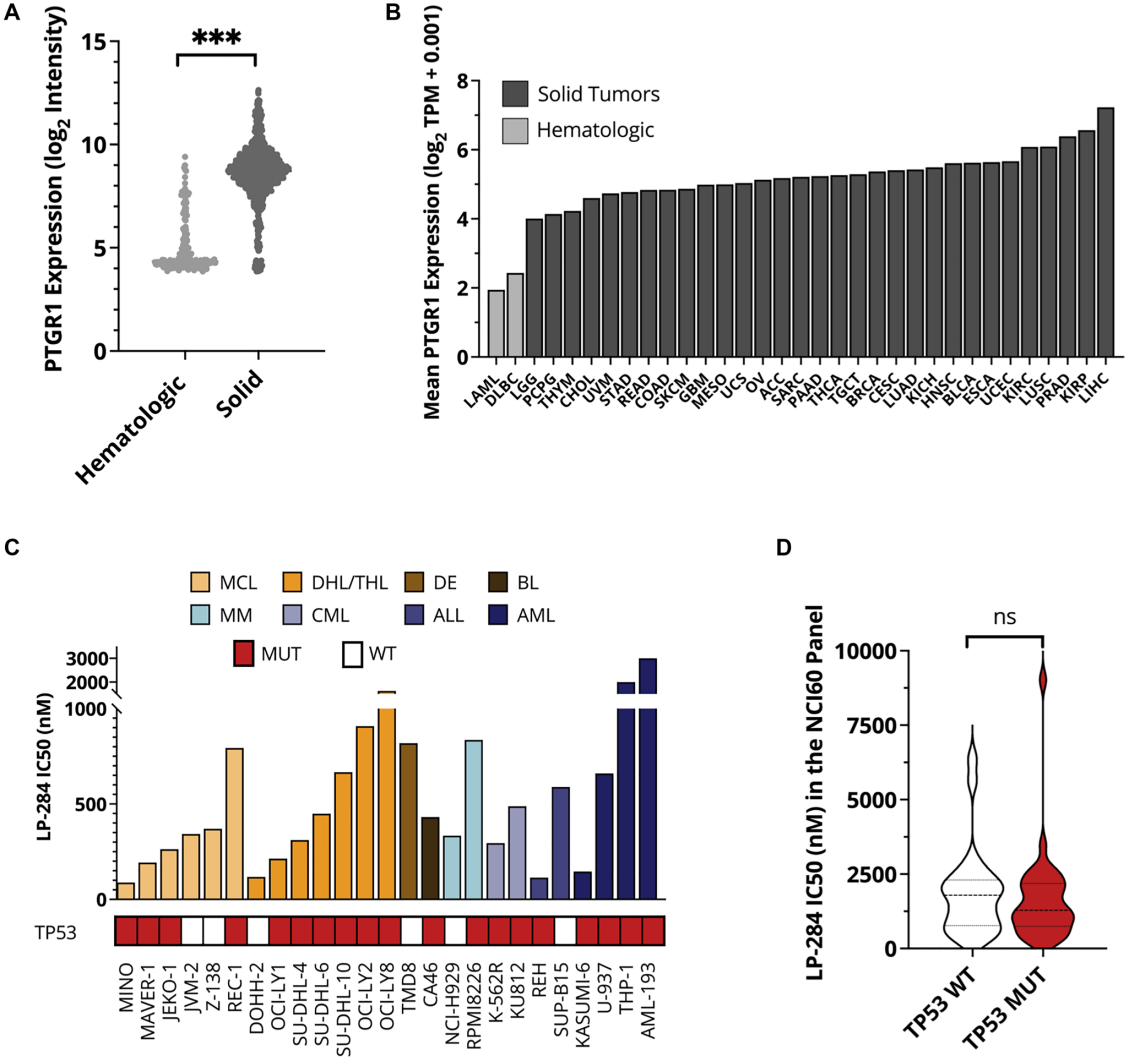
LP-284 exerts robust antitumor activities in hematologic cancer cell lines. (**A**) RNA expression levels of PTGR1 in CCLE hematologic cell lines (*n* = 180) and solid tumor cell lines (*n* = 856). (**B**) Average RNA expression levels of PTGR1 in TCGA patients from 33 cancer types. (**C**) IC50 of LP-284 in a panel of hematologic cell lines measured by 72-hour cell survival assays. (**D**) LP-284 was screened in the full NCI-60 cell panel. IC50 was compared between cells with and without TP53 mutation. Abbreviations: WT: wild type; MUT: mutant; LAML: acute myeloid leukemia; DLBC: diffuse large B-cell lymphoma. Annotations of other TCGA cancer types can be found at https://gdc.cancer.gov/resources-tcga-users/tcga-code-tables/tcga-study-abbreviations.

Previous studies by us and others have demonstrated that both irofulven and LP-184 require PTGR1 for bioactivation [22]. However, the expression level of PTGR1 remains very low in cancer cells of hematologic lineages. We compared PTGR1 RNA expression between 180 hematologic and 856 solid cancer cell lines covered by the cancer cell line encyclopedia (CCLE) [26]. We observed significantly lower expression of PTGR1 in hematologic cancer cell lines with a mean normalized microarray intensity of 4.2 compared to a mean value of 8.7 in solid cancer cell lines (Figure 1A). We also determined that the average expression of PTGR1 is about two-fold lower in primary tumor samples from leukemia and lymphoma patients than those from solid tumor patients in the cancer genome atlas (TCGA) studies (Figure 1B) [27]. Insufficient PTGR1 indicated the limited application of existing acylfulvenes for hematologic cancers. During the process of synthesizing LP-184, we identified its positive enantiomer LP-284. LP-284 was found not to be activated by PTGR1 and had strong anti-tumor activity in the 6 hematologic cancer cell lines in the NCI-60 panel (Zhou et al., 2022 Society of Hematologic Oncology conference abstract # MCL319). It was hypothesized that LP-284 could be applied to hematologic cancer cells with limited PTGR1 expression which has posed a barrier for irofulven and LP-184.

Next, we screened LP-284’s efficacy in a panel of twenty-five cell lines across various hematologic cancer types including MCL, DHL/triple-hit lymphoma (THL), double expressor (DE) lymphoma, Burkitt’s lymphoma (BL), multiple myeloma (MM), CML, acute lymphoblastic leukemia (ALL), and acute myeloid leukemia (AML). The IC50s of these twenty-two of these cell lines, including the imatinib-resistant CML cell line K-562R, were in the nanomolar range (Figure 1C). The average IC50 was 342 nM in the 6 MCL cell lines and 613 nM in the 7 DHL/THL cell lines. TP53 mutation status was not correlated with LP-284 sensitivity in these hematologic cancer cell lines [28–32] (Figure 1C) or in the NCI-60 panel [33] where LP-284’s antitumor activities were screened earlier (Zhou et al., 2022 Society of Hematologic Oncology conference abstract # MCL319) (*p* > 0.05; Figure 1D).

### LP-284 inhibits MCL xenograft growth *in vivo*

We tested LP-284’s *in vivo* efficacy in the JeKo-1 cell line-derived MCL xenograft mouse model. Briefly, mice were implanted with JeKo-1 xenografts and treated with 2 mg/kg or 4 mg/kg body weight of LP-284 (i.v.) every other day for five times in one cycle. LP-284’s efficacy was compared with bortezomib and ibrutinib, whose dosage regimens in xenograft mice were derived from literature [34, 35].

**Figure 2:**
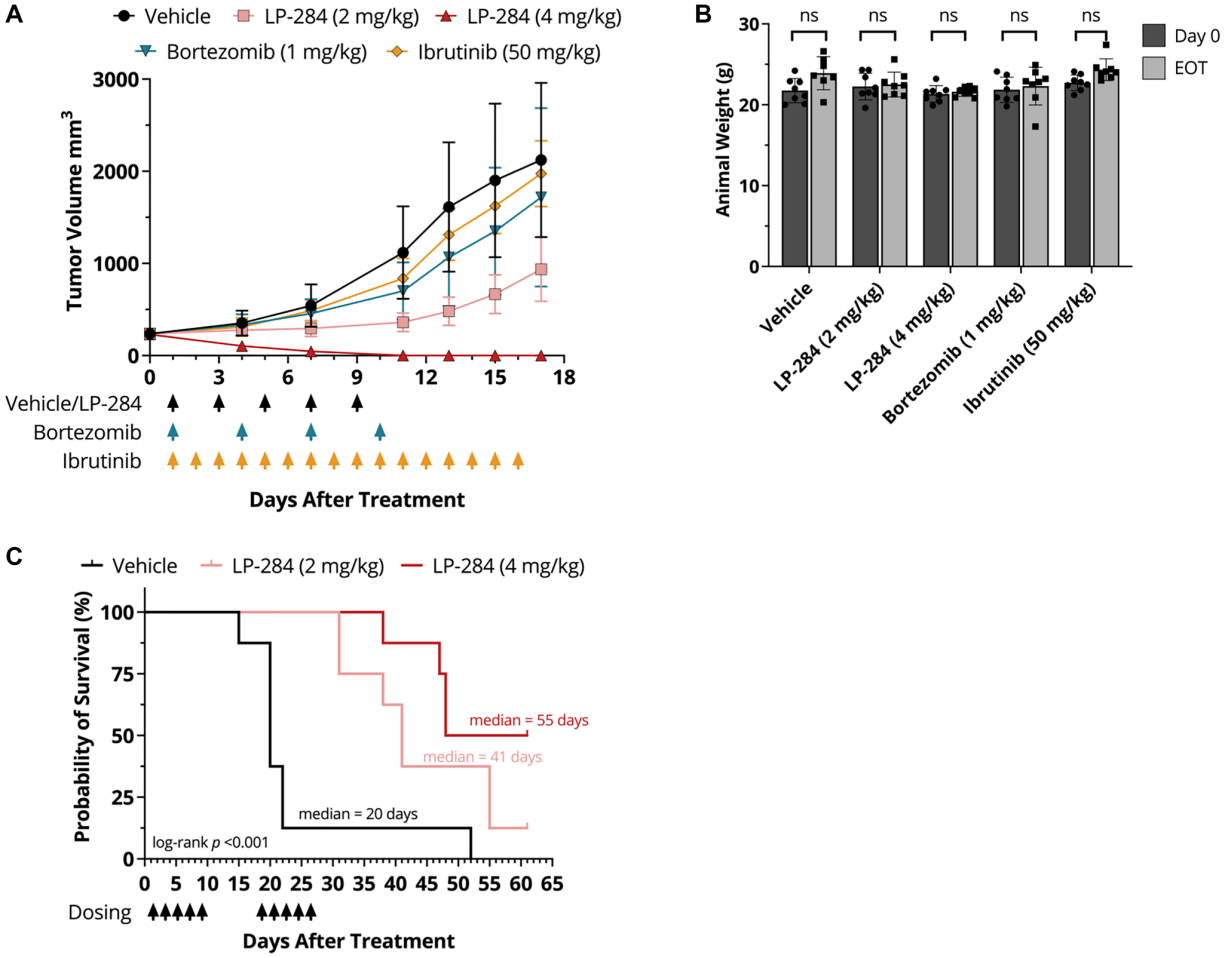
LP-284 outperforms bortezomib and ibrutinib and prolongs the survival of MCL xenograft mice. (**A**) The JeKo-1 MCL cell line-derived xenograft mice (*n* = 8/arm) were treated with vehicle (saline, i.v.), LP-284 (2 mg/kg or 4 mg/kg, i.v.), bortezomib (1 mg/kg, i.p.), or ibrutinib (50 mg/kg, p.o.) at days indicated by arrows in the graph. Tumor volumes were measured every 2~4 days after treatment initiation. Data are represented as mean +/− SD. (**B**) The One-tailed *t*-test was used to examine if the JeKo-1 xenograft mice weighed less on day 17 than on day 0. (**C**) Two cycles of LP-284 and vehicle were administered to JeKo-1 xenograft mice using the regimens shown in the graph. Kaplan-Meier survival curves demonstrated prolonged survival of the LP-284 treated groups. Survival was censored on day 61. Abbreviation: EOT: end of treatment.

At day 17 post-treatment initiation, the percentage of tumor growth inhibition (TGI) in the 4 mg/kg and 2 mg/kg LP-284 arms was 113% and 63%, respectively, and were significantly greater (*p* < 0.05) than the TGIs of bortezomib (22%) and ibrutinib (8%; Figure 2A). No significant weight loss was observed in any of the treatment arms (one-tailed *T*-test, *p* > 0.05; Figure 2B). Furthermore, two treatment cycles of LP-284 significantly prolonged mouse survival by at least two-fold compared to vehicle (saline) (*p* < 0.001; Figure 2C).

### LP-284 diminishes bortezomib-refractory and ibrutinib-refractory MCL xenograft tumors

JeKo-1 MCL xenograft tumors that were refractory to bortezomib and ibrutinib under the dosage regimens tested in Figure 2A had tumor volumes of ~2000 mm^3^ at day 17. The treatment of mice implanted with bortezomib and ibrutinib refractory tumors was then replaced with one cycle of 4 mg/kg LP-284 or the vehicle (saline). Bortezomib and ibrutinib refractory mice were stratified based on their tumor size into two groups of four mice each that were then treated by LP-284 or the vehicle.

**Figure 3:**
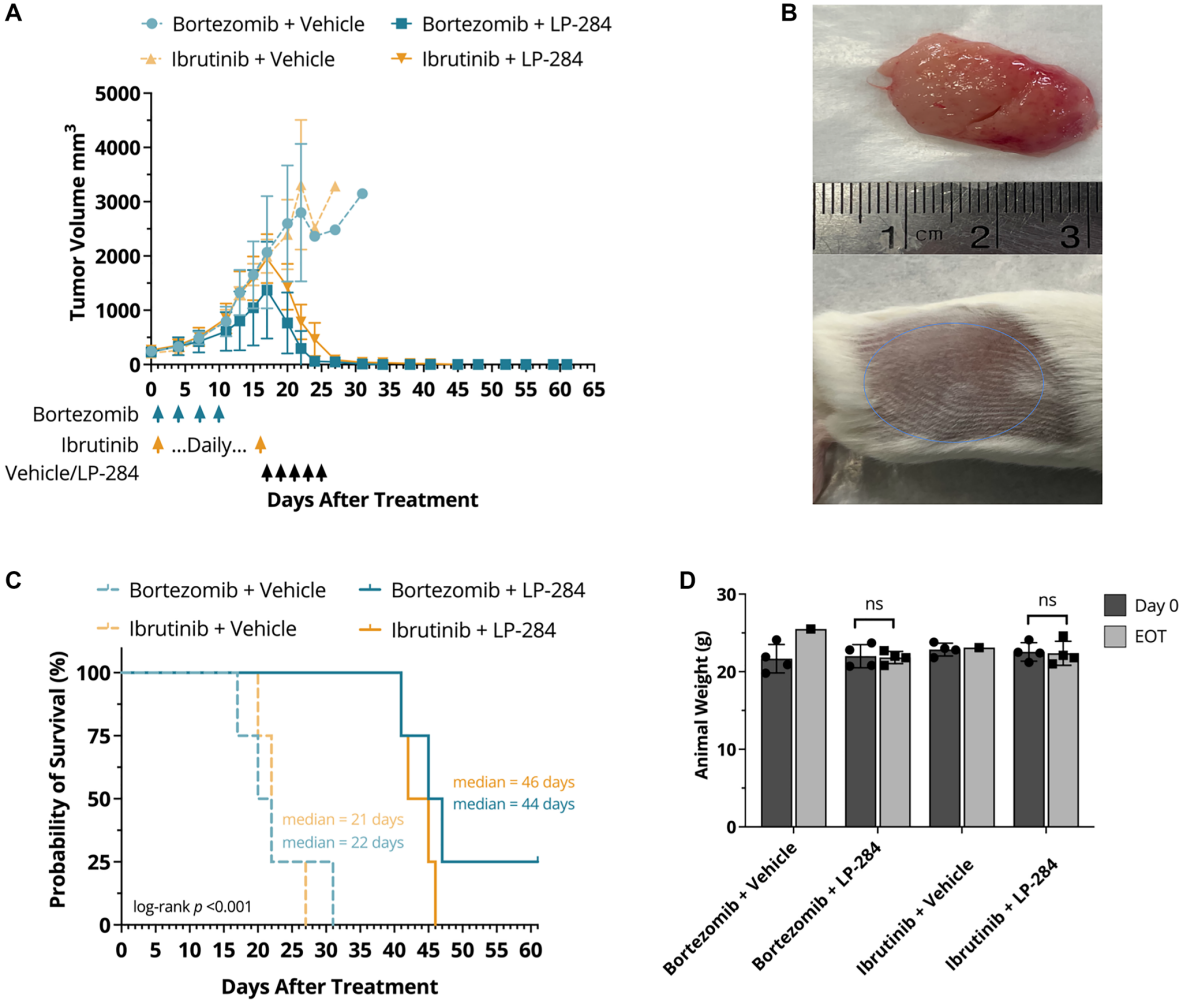
LP-284 rescues bortezomib-refractory and ibrutinib-refractory MCL xenograft mice. The JeKo-1 MCL cell line-derived xenograft mice were treated with bortezomib (1 mg/kg, i.p.) or ibrutinib (50 mg/kg, p.o.) till day 16 when the tumor sizes reached ~2000 mm^3^. Each treatment arm was then further split into two sub-arms (*n* = 4/sub-arm), one of which was treated by vehicle (saline) whereas the other was treated by 4 mg/kg LP-284 (i.v.), as indicated by arrows in the graph. (**A**) Tumor volumes decreased rapidly in the LP-284 arms. Data are represented as mean +/− SD. (**B**) Representative photos show large tumors in one mouse after switching to vehicle treatment (upper) and near complete tumor regression in one mouse after switching to LP-284 treatment (lower). (**C**) Kaplan-Meier survival curves demonstrated prolonged survival of the LP-284 rescued groups. Survival was censored on day 61. (**D**) Animal weight comparison at day 0 and EOT. Abbreviation: EOT: end of treatment.

One cycle of 4 mg/kg LP-284 led to near-complete tumor regression, whereas the vehicle-treated tumors continued to grow until the mice died or were sacrificed due to excess tumor size (Figure 3A and 3B). In addition, LP-284 resulted in ~2X longer survival (*p* < 0.001) than vehicle treatment in the bortezomib or ibrutinib pre-treated and refractory xenograft mice (Figure 3C). No significant weight loss was observed at the end of treatment (EOT) in any of the treatment arms (one-tailed *T*-test, *p* > 0.05; Figure 3D).

### LP-284 induces HR response, cell apoptosis, and DSB

**Figure 4:**
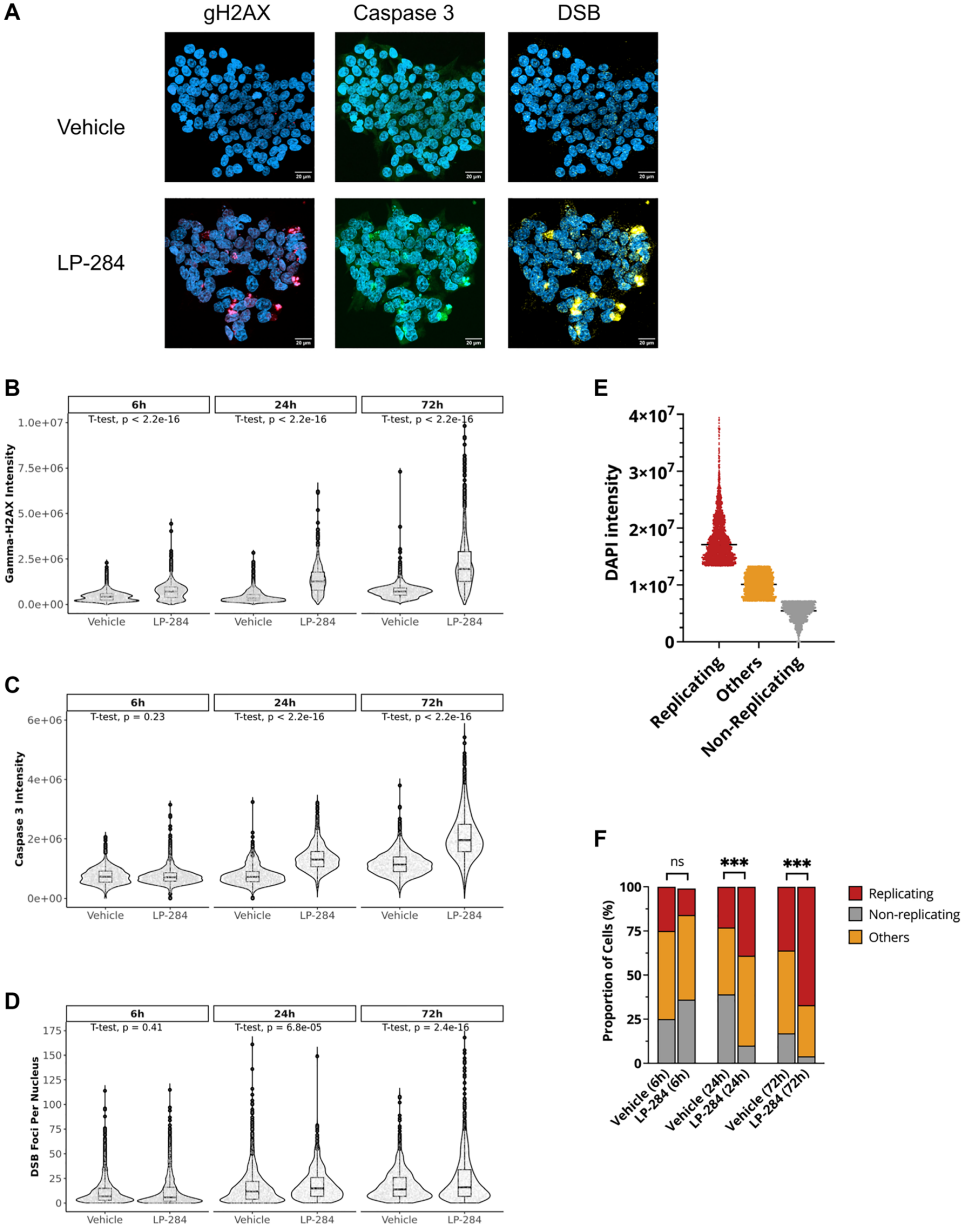
LP-284 induces H2AX phosphorylation, cell apoptosis, and DSBs. The leukemia cell line HAP1 was treated with vehicle (DMSO) or LP-284 at IC50 for 6, 24, and 72 hours. A total of 463,123 nuclei in the vehicle group and 316,704 nuclei in the LP-284 group were surveyed. Cells were stained with DAPI (blue), gamma-h2ax (gH2AX, Ser139, red), and cleaved caspase 3 (green). The amount of DSB foci (yellow) per nucleus was directly detected and quantified using the double strand-DNA breaks with STRIDE (dSTRIDE) technology. (**A**) Representative images of gH2AX, caspase 3, and DSB staining. (**B**) Comparison of gH2AX intensity between vehicle and LP-284 treated cells over time. (**C**) Comparison of cleaved caspase 3 intensity between vehicle and LP-284 treated cells over time. (**D**) Comparison of the number of DSB foci per nucleus between vehicle and LP-284 treated cells over time. One-tailed student’s *t*-test was performed for 2 sample comparisons. (**E**) Cells were grouped into replicating (top 25%), non-replicating (bottom 25%), or others based on DAPI intensity in cells treated by the vehicle for 6 hours. (**F**) The proportion of cells at different stages. Two-proportions *Z*-test was performed to compare the proportion of replicating cells between LP-284 and vehicle-treated samples. ^*^ *p* < 0.05; ^**^ *p* < 0.01; ^***^ *p* < 0.001; Abbreviation: ns: not significant.

To determine the mechanism of action of LP-284’s potent antitumor activity, we examined LP-284-induced damage at the cellular level. The leukemia cell line HAP1 was treated with vehicle (DMSO) or 850 nM LP-284 for 6, 24, and 72 hours and stained with phosphorylated H2AX (gH2AX) to monitor HR response and cleaved caspase 3 to monitor apoptosis (*n* = 2 per group). We also quantified the number of DSB foci with the SensiTive Recognition of Individual DNA Ends (STRIDE) assay (Figure 4A), a novel and highly sensitive technique that allows specific and direct detection of DNA breaks, such as DSBs, in the nucleus of single cells [36].

A total of 463,123 nuclei in the vehicle group and 316,704 nuclei in the LP-284 group were surveyed. LP-284-induced gH2AX was significantly different at 6 hours post-treatment when compared with the vehicle-treated cells (one-tailed *T*-test, *p* < 2.2e-16) and increased over time (Figure 4B). At both 24 and 72 hours, LP-284 treated cells had around 3X higher mean intensity of gH2AX than vehicle-treated cells (one-tailed *T*-test, *p* < 2.2e-16). Elevated caspase 3 intensity was not observed at 6 hours (one-tailed *T*-test, *p* = 0.23), but became significant at 24 and 72 hours (one-tailed *T*-test, *p* < 2.2e-16; Figure 4C). Though HAP1 cells are thought to maintain an integral DDR system [37], we speculated that prolonged treatment of LP-284 would still result in some cells with an increased number of DSB foci. As shown in Figure 4D, the majority of nuclei treated by either the vehicle or LP-284 had fewer than 50 DSB foci per nucleus at 6 hours post-treatment and no significant difference was found between these two groups (*p* = 0.41). At 24 hours, the LP-284 treated cells exhibited a slightly increased mean number of DSB foci per nucleus (one-tailed *T*-test, *p* = 6.8e-05). At 72 hours post-treatment, 15% of LP-284-treated cells whereas only 3% of vehicle-treated cells had more than 50 DSB foci per nucleus (two-proportions Z-test, *p* < 2.2e-16).

Cell cycle analysis was performed based on the intensity of DAPI staining. Using the DAPI intensity in the 6-hour vehicle-treated cells as the classifier, we considered cells with DAPI intensity in the top 25 percentile as replicating cells (late S or G2) and the ones in the bottom 25 percentile as non-replicating cells (Figure 4E). At 6 hours post-treatment, there were no differences in the proportion of replicating cells between the vehicle and LP-284 group (two-proportions *Z*-test, *p* > 0.05). However, LP-284 treatment resulted in a greater proportion of replicating cells after 24 and 72 hours (two-proportions *Z*-test, *p* > 0.05; Figure 4F).

### Increased sensitivity to LP-284 in ATM-deficient cells

**Figure 5:**
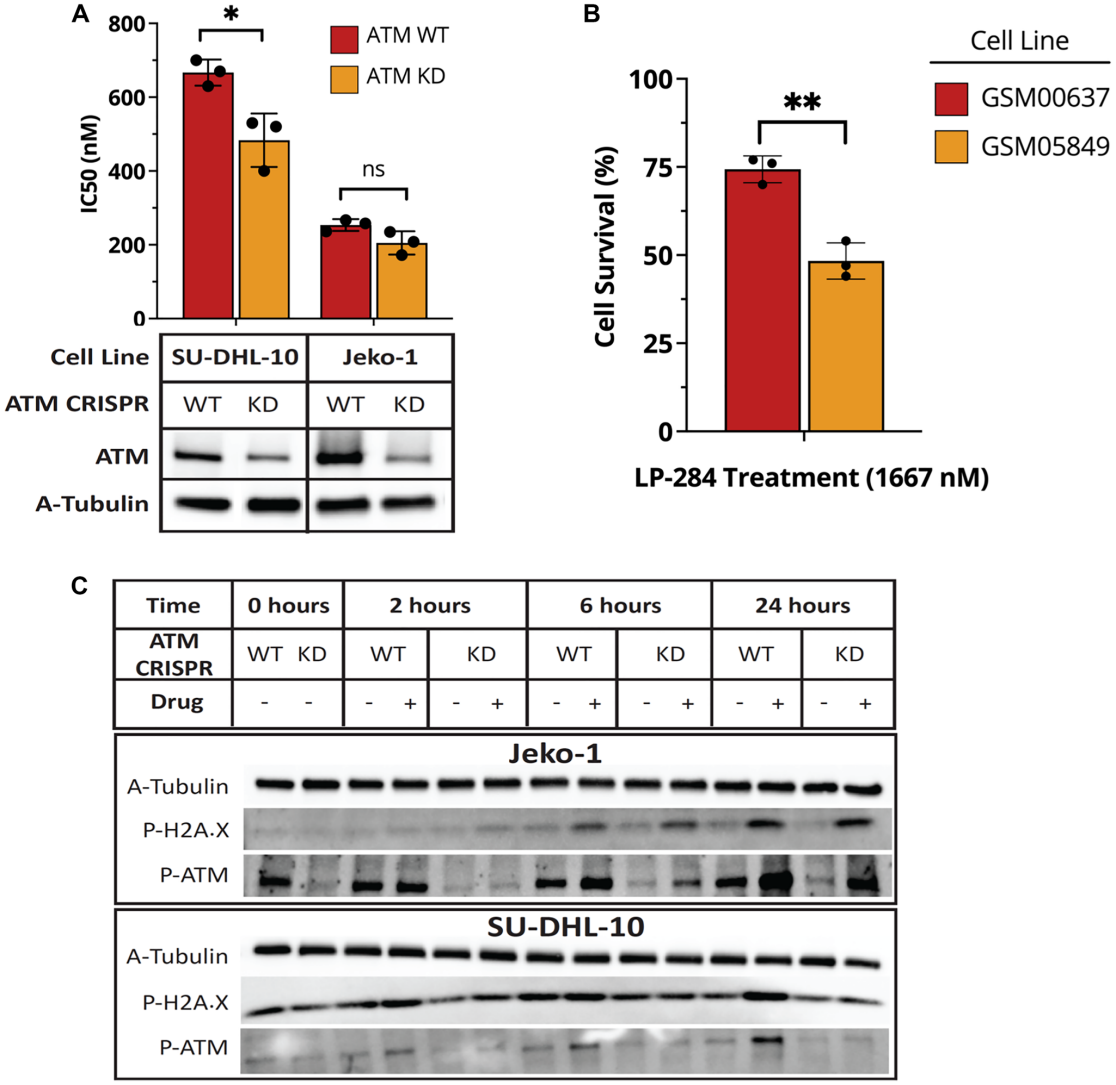
ATM-deficient cells show increased sensitivity to LP-284. (**A**) The ATM gene was knocked down in the DHL cell line SU-DHL-10 and the MCL cell line JeKo-1. Western blots were performed to confirm the reduced protein expression of ATM. Cell viability in WT and KD cell lines were measured 72 hours after LP-284 treatment and IC50 values were compared by Student’s *t*-test. (**B**) GM00638, and GM05849 are ATM-proficient and deficient fibroblast cells, respectively. They were treated with 1667 nM LP-284 for 72 hours and cell viability was measured. (**C**) Phosphorylation of H2AX and ATM in SU-DHL-10 and JeKo-1 were measured by western blots at 0, 2, 6, and 24 hours after treating by LP-284 at ½ IC50. ^*^ *p* < 0.05; ^**^ *p* < 0.01; Abbreviation: ns: not significant.

Given the effects of LP-284 on both HR and DSB, we hypothesized that cells with reduced ATM expression would be unable to efficiently activate HR repair rendering them more sensitive to LP-284-induced DSB. Accordingly, we knocked down the ATM gene in the DHL cell line SU-DHL-10 and the MCL cell line JeKo-1 using the CRISPR-Cas9 approach. We confirmed the reduction of ATM protein level by western blot analysis. As shown in Figure 5A, the reduction of ATM expression was associated with a significant reduction in LP-284’s 72-hour IC50 in SU-DHL-10 (*p* < 0.05; Figure 5A). Consistently, in the SU-DHL-10 WT cells, we observed activated HR as indicated by increased ATM phosphorylation and gH2AX after exposure to either vehicle or LP-284. However, in the SU-DHL-10 KD cells, there was no sign of HR activation with comparable levels of phosphorylated ATM or gH2AX between the vehicle and LP-284 treated cells (Figure 5C). The JeKo-1 ATM KD cells exhibited lower though not statistically significant IC50 compared to its WT counterpart (Figure 5A). Consistent with the observation, we observed LP-284-induced HR, indicated by ATM phosphorylation and gH2AX, in both JeKo-1 WT and KD cells at both 6 hours and 24 hours post-treatment (Figure 5C). We also explored the correlation between ATM and LP-284 response in a pair of non-tumor fibroblast cells with well-characterized ATM status [38]. Treatment of 1667 nM LP-284 led to an average of 56% cell survival in the ATM-deficient GSM05849, which is significantly lower than the average of 73% cell survival in the ATM-proficient GSM00637 (*p* < 0.01; Figure 5B).

### Inhibition of TC-NER sensitizes cells to LP-284

**Figure 6:**
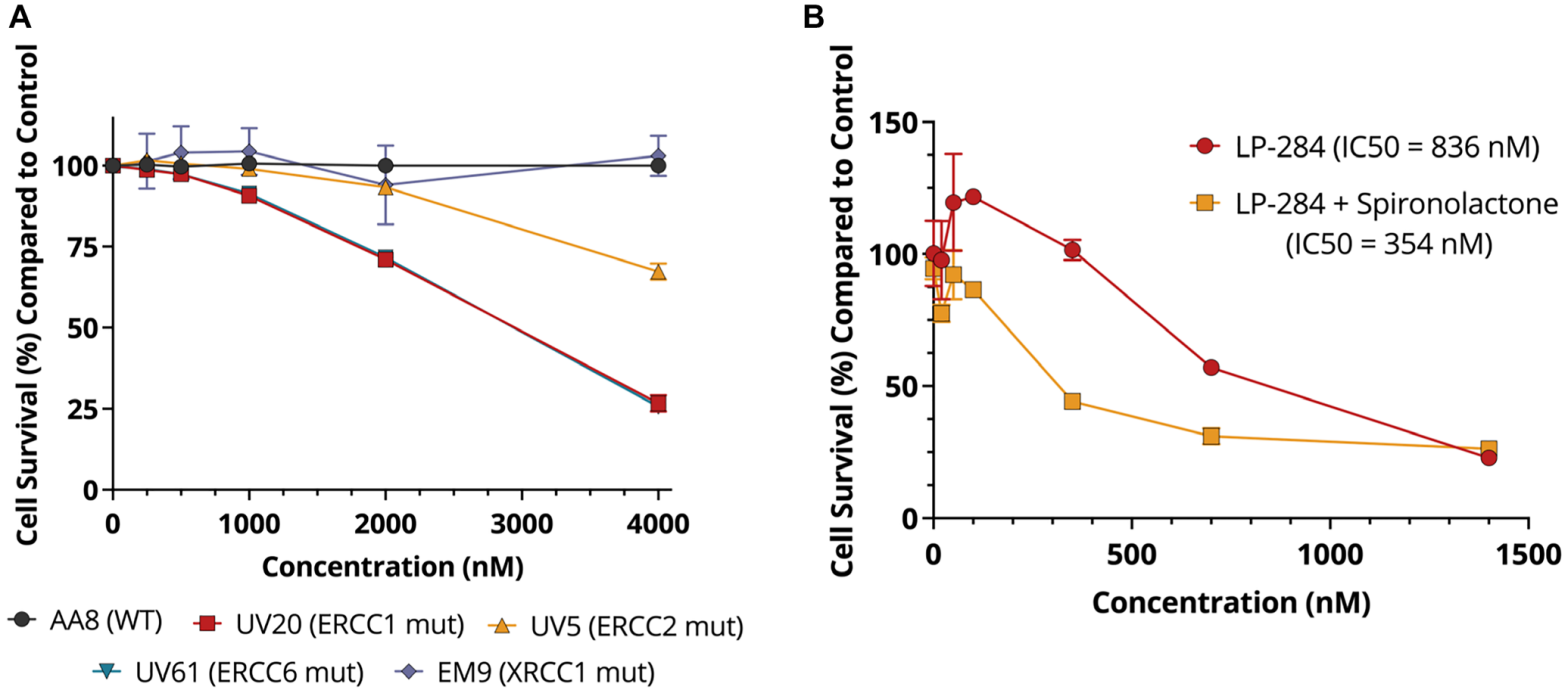
LP-284 is selectively lethal in cells with TC-NER deficiency. (**A**) CHO wild-type (WT) cells, isogenic mutant cells carrying TC-NER mutations (ERCC1, ERCC2, or ERCC6), and BER mutation (XRCC1) were treated by LP-284 for 72 hours in triplicates. Cell survival was measured by Alamar Blue assays. (**B**) The multiple myeloma cell RPMI8226 was treated by LP-284 with and without the combination of a non-toxic dose of spironolactone (10 μM) for 24 hours in triplicates. Data are represented as mean +/− SD.

As acylfulvenes are known to induce TC-NER lesions [20, 21], we studied the toxicity of LP-284 in well-characterized Chinese hamster ovary (CHO) cells with defects in TC-NER [39] and BER [40]. In CHO cells carrying mutations in the TC-NER core genes ERCC1, ERCC2, or ERCC6, cell survival decreased with increased LP-284 dose, resulting in 26%~67% cell survival compared with the vehicle (DMSO) treated WT cells at the highest dose (4 μM) used in the study (one-tailed *T*-test, *p* < 0.001). However, no changes in cell survival were observed in the parent WT cell line and the one carrying the BER mutation XRCC1 (one-tailed *T*-test, *p* = 0.753; Figure 6A). We further validated the synthetic lethal relationship between TC-NER deficiency and sensitivity to LP-284 through pharmacological inhibition of TC-NER. Spironolactone is an FDA-approved drug for treating hypertension and also possesses the ability to degrade the key TC-NER gene ERCC3 [41]. It is known that adding 10 μM spironolactone to the MM cell line RPMI8226 does not reduce cell viability, but results in the degradation of ERCC3 after 24 hours [42]. Using the same condition, we found that spironolactone alone didn’t cause cell cytotoxicity but spironolactone in combination with LP-284 led to a 2.4-fold decrease (*p* < 0.05) in LP-284’s 24-hour IC50 in the same RPMI8226 cell line (Figure 6B).

## DISCUSSION

In the current study, we demonstrated the new acylfulvene compound LP-284 has anti-tumor activity including nanomolar potency in fifteen *in vitro* NHL cell lines and *in vivo* preclinical NHL models. In the *in vivo* MCL xenograft studies, LP-284 was demonstrated to have increased TGI when compared to bortezomib and ibrutinib and is capable of prolonging the survival of xenograft mice by more than 2X (Figure 2). Bortezomib, a proteasome inhibitor, and ibrutinib, a Bruton’s tyrosine kinase (BTK) inhibitor, have been widely used in NHL, but cures are rarely achieved. In comparison with bortezomib and ibrutinib, LP-284 displays at least three times stronger TGI (Figure 2). Furthermore and more importantly, in the same study, LP-284 can almost completely eliminate those NHL xenograft tumors that are refractory to bortezomib or ibrutinib (Figure 3). There are numerous resistant mechanisms of these two drugs such as mutation and deregulation of the proteasome subunits, BTK mutation, upregulated NF-*κ*B, and cell survival pathways [43]. LP-284 is likely able to overcome bortezomib and ibrutinib resistance due to its entirely different mechanism of action on targeting DDR deficiencies. Our results suggest that LP-284 treatment may be a viable salvage treatment plan for patients who are refractory to bortezomib or ibrutinib.

Dysregulation of DDR is a common phenomenon in NHL cells [6]. There are at least two approaches to utilizing DDR deficiency as a therapeutic target. One approach is to target the remaining functional DDR pathways with DDR inhibitors, like PARP inhibitors (PARPi), preventing cells from repairing endogenous DNA damage that occurs constantly. PARPi block the repair of single-strand DNA break, leading to the generation of DSBs that are synthetic lethal in HR-deficient cells. PARPi has been successfully used as a targeted therapy in some solid tumors and is now being explored in the treatment of hematologic malignancies [44]. Another approach is to introduce DNA damage in cells that cannot repair such lesions. Since non-tumor cells typically maintain fully-functional DDR, this approach provides a precise killing mechanism for DDR-deficient tumors. We used the quantitative STRIDE technology to assess the number of LP-284-induced DSB foci at the resolution of a single nucleus in HAP1 cells. Increased number of cells with accumulated DSB was noticeable at 24 hours post-treatment and peaked at 72 hours (Figure 4). HAP1 doesn’t have mutations in any of the major DDR pathway genes [37]. It is possible that some of the induced DSBs were being repaired by HR in HAP1 cells during the treatment course, as we have detected increased gH2AX at 6 hours post-treatment. Interestingly, we found that LP-284 treatment led to an increased proportion of replicating cells (late S or G2). As HR repair takes place in S or G2 phases, it is possible that the arrested cells at S or G2 are due to ongoing HR repair of LP-284-induced DSBs. Given these observations, we hypothesize that LP-284 induces DSBs that can be repaired by functional HR mechanisms.

ATM plays a central role in HR and is also the most frequently deregulated gene in MCL [13]. Camacho et al. reported that seven out of the seventeen MCL tumor specimens examined had absent or very low ATM protein expression [45]. We found a 1.4-fold increase in sensitivity to LP-284 when the ATM gene was knocked down in SU-DHL-10 (*p* < 0.05). We think this is driven by the disrupted HR as no induction of phosphorylation of ATM nor H2AX was noticeable in the SU-DHL-10 ATM KD cells (Figure 5A). We also observed significantly increased sensitivity to LP-284 in ATM-deficient fibroblast cells compared to its ATM-proficient counterparts (Figure 5B), which is possibly driven by the dysfunctional HR as well. Interestingly, only a mild increase of sensitivity to LP-284 was observed in the JeKo-1 ATM KD cells, which could be due to the still viable HR as evidenced by the phosphorylation of ATM and H2AX. (Figure 5A and 5C). Though the protein expression level of ATM is comparable between SU-DHL-10 KD and JeKo-1 KD, it is likely that the threshold level for ATM to elicit DSB repair is different in these two cell lines. We hypothesize that a more significant difference in IC50 can be observed if ATM is completely knocked down or if the ATM function is compromised due to mutations.

As failure to repair DSB or HR deficiency increases NHL cells’ sensitivity to LP-284, targeting HR-deficient NHL patients with LP-284 is likely to produce better treatment outcomes. In addition to ATM, HR deficiency has also been identified in NHL cells carrying other genetic abnormalities. Expression of the transcription factor LMO2 in more than half of DLBCL patients is thought to prevent the recruitment of BRCA1, leading to the BRCAness phenotype and hypersensitivity to PARPi. Mutation or overexpression of EZH2 inhibits HR and enhances apoptosis from DNA-damaging agents. The anti-apoptotic protein BCL2 is also known to decrease DDR [6]. IGH/MYC translocation is also associated with BRCA2 deficiency [46]. Therefore, there is likely to be a broad range of NHL patients with HR deficiency who may benefit from DSB-inducing agents such as LP-284.

TP53 mutation is typically associated with poor prognosis in NHL patients. In our preclinical models, we did not observe any correlation between TP53 mutation and LP-284’s antitumor activities. The JeKo-1 model used in our xenograft study contains a lethal TP53 deletion (p.P58fs) and it responds well to LP-284. The finding of TP53-independence is consistent with the one made by Poindessous et al. [25] on LP-284’s acylfulvene analog Irofulven. In Poindesous’ study, the cytotoxicity of irofulven is not affected by the loss of TP53 function. In the same study, they also reported that irofulven has a very different anti-tumor spectrum from cisplatin and ET-743 [25]. Similarly, we found that LP-284 displays a unique profile of antitumor activities among the FDA-approved alkylating agents for hematologic malignancy treatment (Supplementary Figure 1). It is possible that LP-284 would not exhibit cross-resistance with conventional alkylating agents and thus patients that are refractory to the commonly used therapies such as bendamustine and R-CHOP would still be suitable for LP-284 treatment.

In conclusion, our study has demonstrated LP-284 as a novel and potent acylfulvene drug that can suppress tumor growth in NHL models and cells with HR or TC-NER deficiency. LP-284 has promising potential to treat patients that are refractory to BTK or proteasome inhibitors and especially those with unfavorable genetic features such as ATM, TP53, and double translocations of MYC/BCLs. Further identification and validation of LP-284’s biomarkers would facilitate the delivery of LP-284 as a targeted approach towards NHL patients with defective HR or TC-NER.

## MATERIALS AND METHODS

### Cell lines and chemicals

The following cell lines were purchased from the American Type Culture Collection (ATCC): K-562R, THP-1, KASUMI-6, AML-193, SUP-B15, KU-812, U-937, CA46, SU-DHL-6, REH, NCI-H929, JVM-2, JEKO-1, MAVER-1, MINO, REC-1, Z-138, RPMI8226, SU-DHL-4, and SU-DHL-10. OCI-LY1 was purchased from the German Collection of Microorganisms and Cell Cultures GmbH (DSMZ). DOHH-2, OCI-LY2, OCI-LY-8, TMD8, HAP1, AA8, UV5, UV20, UV21, and EM9 were available at and authenticated by our contract research organizations. Human fibroblast cells GM00637 and GM05849 were purchased from Coriell Institute for Medical Research. All the cell lines were cultured in medium according to the vendors’ recommendation in cell culture incubators set at 37°C with 5% CO2. LP-284 was provided by Lantern Pharma Inc., and stored at −80°C. LP-284 was formulated from powder by dissolving in 5% EtOH and 95% saline (*in vivo* studies) or DMSO (*in vitro* studies). Bortezomib was purchased from MedChemExpress (Cat. HY-10227) and formulated with 1% DMSO and 99% Saline. Ibrutinib was purchased from MedChemExpress (Cat. HY-10997) and formulated with 10% DMSO, 40% PEG 300, 5% Tween 80, and 45% saline. Spironolactone was purchased from Selleckchem (Catalog No.S4054) and formulated with 100% DMSO.

### Cell viability assay

Cell viability assay was conducted using the CellTiter-Fluor™ Cell Viability Assay (Progema, G6082) for cell lines if not specified below. The viabilities of SU-DHL-10, OCI-LY1, OCI-LY2, OCI-LY8, and TMD-8 were measured by the 3-(4,5-dimethylthiazol-2-yl)-2,5-diphenyl-2H-tetrazolium bromide (MTT) assay according to the manufacturer’s recommendation (VWR, 103258-310). The viability of CHO cells was measured by the AlamarBlue assay. Cells (4000~8000/well) were seeded in 96 well plates and treated with vehicles (DMSO) or various doses of LP-284 for 72 hours in triplicates at 37°C with 5% CO_2_. For CellTiter-Fluor™ assays, after 72 hours of total incubation, 50 μl of GF-AFC Reagent (10 μl of GF-AFC substrate diluted in 5 ml of assay buffer for each 96 well plate) was added to each well, mixed, and incubated for 30 minutes at 37°C. For the MTT assay, after treatment, 5 mg/ml MTT solution was added to each well, mixed, and incubated at 37°C for 4 hours before the addition of the stopping buffer. Cells were incubated for another 24 hours before cell viability assays. For the Alamar Blue assay, immediately after the exposure time was reached, the Alamar Blue solution in fresh cell culture medium was added to the wells to a final concentration of 0.001%. Cells were incubated for another 3 hours. The absorbance was measured using a microplate reader and data was collected for further analysis.

### LP-284 and spironolactone combination assay

Around 15,000 RPMI-8226 cells with 40 μl of growth medium were seeded in each well of a tissue culture-treated flat bottom black-sided 96 well plates. Cell culture medium containing spironolactone or vehicle (DMSO) was added to a concentration of spironolactone at 10 μM. After 15~30 minutes of pre-incubation, 50 μl of spironolactone, spironolactone + LP-284, LP-284, or vehicle were added accordingly to reach 100 μl in each well. Cells were incubated for another 24 hours before CellTiter-Fluor™ Cell Viability Assay (Progema, G6082).

### Generation of ATM knock-down cells

ATM gene knockdowns in JeKo-1 and SU-DHL-10 were performed using the CRISPR/CAS9 method. The SgRNA sequences used were CACCGGTGAAATATCTCAGCAACAG and AAACCTGTTGCTGAGATATTTCACC. The oligo was ligated into the pSpCas9(BB)-2A-GFP (PX458) (Addgene, 48138) to make the knockdown construct. The construct was then transfected into cells via nucleofection. Successfully transfected cells were selected by single-cell sorting (Sony, SH800) based on GFP positivity. Single cells were expanded for 2 to 3 weeks. DNA was isolated using the Nucleospin Tissue kit (Macherey-Nagel, 740952.50). The DNA was then subjected to PCR amplification using primers outside the sgRNA region (Forward: ATTCATCTAATGGTGCTATTTACGG and Reverse: GTTAAACTGTCAGGTCACTTGGG). Confirmation of ATM INDEL events was validated by Sanger sequencing of PCR-amplified DNA.

### SensiTive recognition of individual DNA ends (STRIDE) assay and detection of gamma-H2AX and apoptosis

HAP1 cells were seeded on cover glasses in 12-well plates at a density of 16,000 cells/well and left to rest for 24 hours. Next, cells were treated with vehicle (DMSO) or 850 nM LP-284 for 6, 24, and 72 hours. After treatment, all samples were fixed with ice-cold 70% EtOH and stored at −20°C. STRIDE assay to detect DSB foci was performed as described in Kordon et al. [36]. Cells were then counterstained with DAPI (Thermo Fisher, D1306), phospho-H2A.X (Ser139) (1:100 dilution, Alexa Fluor^®^ 647, Cell Signaling Technology, 9720), and cleaved Caspase-3 (Asp175) (1:50 dilution, Alexa Fluor^®^ 488, Cell Signaling Technology, 9603) for 90 min. The coverslips with fixed cells were then mounted with Vectashield (Vector Laboratories) and stored at 4°C before imaging. Fields of view for imaging (at least 10 per sample) were chosen randomly over the surface of the coverslip. The images were then collected as 3D confocal stacks using Cell Discoverer 7 LSM 900 microscope, Zeiss. Quantification of DSB foci was performed as described in Kordon et al. [36].

### Western blot

Cells were collected and lysed using RIPA buffer supplemented with a protease inhibitor cocktail. Lysates were loaded onto SDS-PAGE gels and ran at 150 V for 1.5 hours. Proteins were then transferred to PVDF membranes and blocked with TBST (Tris-buffered saline and 0.05% Tween 20) supplemented with 5% nonfat milk. The membranes were blotted with the following primary antibodies overnight at 4°C: ATM (Cell Signaling, 2873S, 1:1,000 dilution), p-ATM (Cell Signaling, 13050S, 1:1,000 dilution), p-H2AX (Cell Signaling, 2557S, 1:1000 dilution), and Tubulin (Cell Signaling, 2128S, 1:1,000 dilution). After washing with TBST, the membranes were incubated with the secondary antibodies at room temperature for 1 hour: anti-mouse or rabbit IgG HRP-linked (Cell Signaling, 7076S or 7074S respectively, 1:10,000 dilution). Tubulin was used as a control of protein loading.

### *In vivo* animal study

Five-week-old NOD.SCID mice were procured through Envigo (Item 17003F). Mice were fed Teklad irradiated (sterilized) mouse diet and bedded with Teklad irradiated (sterilized) corncob bedding from Envigo (Indianapolis, IN, USA). Mice were housed in Optimice carousel sterile quarters with a filtered air supply in disposable cages from Animal Care Systems, Inc. (Centennial, CO). A total of 1 × 10^7^ cells JeKo-1 cells were injected subcutaneously into the right hind flank of each animal using a 27-gram needle. Vehicle (saline), 4 mg/kg or 2 mg/kg LP-284 was administered as an intravenous injection on days 1, 3, 5, 7, 9, 17, 19, 21, 23, 25. 50 mg/kg ibrutinib was administered orally daily on days 1–16. 1 mg/kg bortezomib was administered as an intraperitoneal injection on days 1, 4, 7, and 10. The ibrutinib and bortezomib arms were further treated with vehicle or 4 mg/kg LP-284 intravenously on days 17, 19, 21, 23, and 25. Tumors were measured in two dimensions using calipers, and volume was calculated using the formula:


$$\text{Tumor Volume }({\text{mm}}^{3})=w^{2}\times l\text{/}2$$

where *w* = width and *l* = length, in mm, of the tumor. For this study, the calipers were aligned to the tumor edges (the tumors were not squeezed with the caliper).

### Statistical analysis

Statistical analysis was conducted using GraphPad PRISM^®^ (versions 9.0, GraphPad Software, Inc., USA) software. Student’s *t*-test was used for two group comparisons. Two-proportion *Z*-test was used for proportion comparisons. Statistical significance was evaluated by *p* values (ns: *p* ≥ 0.05; ^*^ *p* < 0.05; ^**^ *p* < 0.01, ^***^ *p* < 0.001). The log-rank test was conducted for the survival study and Kaplan-Meier survival curves were generated.

## SUPPLEMENTARY MATERIALS



## Abbreviations

ALL: acute lymphocytic leukemia
AML: acute myeloid leukemia
ATM: ataxia-telangiectasia mutated
BER: base excision repair
BL: Burkitt’s lymphoma
BRCA1: breast cancer gene 1
BTK: Bruton’s tyrosine kinase
CCLE: cancer cell line encyclopedia
CHO: Chinese hamster ovary
CLL: chronic lymphocytic leukemia
CML: chronic myeloid leukemia
DDR: DNA damage repair
DE: double expressor
DHL: double-hit lymphoma
DLBCL: diffuse large B cell lymphoma
DSB: double-strand break
EOT: end of treatment
GG-NER: global genome nucleotide excision repair
gH2AX: gamma-H2AX
H2AX: H2A.X Variant Histone
HR: homologous recombination
HRD: homologous recombination deficient
KD: knock-down
MCL: mantle cell lymphoma
MMR: mismatch repair
MoA: mechanism of action
NHL: non-Hodgkin’s lymphoma
PARP: poly-ADP ribose polymerase
PARPi: PARP inhibitors
PTGR1: prostaglandin reductase 1
SLL: small lymphocytic leukemia
STRIDE: SensiTive recognition of individual DNA ends
TCGA: the cancer genome atlas
TC-NER: transcription-coupled nucleotide excision repair
THL: triple-hit lymphoma
WT: wild type

## References

[1] Thandra KC , Barsouk A , Saginala K , Padala SA , Barsouk A , Rawla P . Epidemiology of Non-Hodgkin’s Lymphoma. Med Sci (Basel). 2021; 9:5. 10.3390/medsci9010005. 33573146 PMC7930980

[2] Burkart M , Karmali R . Relapsed/Refractory Mantle Cell Lymphoma: Beyond BTK Inhibitors. J Pers Med. 2022; 12:376. 10.3390/jpm12030376. 35330376 PMC8954159

[3] Kesireddy M , Lunning M . Relapsed or Refractory Diffuse Large B-Cell Lymphoma: “Dazed and Confused”. Oncology (Williston Park). 2022; 36:366–75. 10.46883/2022.25920963. 35723942

[4] Kumar A , Eyre TA , Lewis KL , Thompson MC , Cheah CY . New Directions for Mantle Cell Lymphoma in 2022. Am Soc Clin Oncol Educ Book. 2022; 42:1–15. 10.1200/EDBK_349509. 35561299

[5] Laude MC , Lebras L , Sesques P , Ghesquieres H , Favre S , Bouabdallah K , Croizier C , Guieze R , Drieu La Rochelle L , Gyan E , Chin R , Aurran-Schleinitz T , Marouf A , et al. First-line treatment of double-hit and triple-hit lymphomas: Survival and tolerance data from a retrospective multicenter French study. Am J Hematol. 2021; 96:302–11. 10.1002/ajh.26068. 33306213

[6] Carrassa L , Colombo I , Damia G , Bertoni F . Targeting the DNA damage response for patients with lymphoma: Preclinical and clinical evidences. Cancer Treat Rev. 2020; 90:102090. 10.1016/j.ctrv.2020.102090. 32892059

[7] Bakr A , Oing C , Köcher S , Borgmann K , Dornreiter I , Petersen C , Dikomey E , Mansour WY . Involvement of ATM in homologous recombination after end resection and RAD51 nucleofilament formation. Nucleic Acids Res. 2015; 43:3154–66. 10.1093/nar/gkv160. 25753674 PMC4381069

[8] Balmus G , Pilger D , Coates J , Demir M , Sczaniecka-Clift M , Barros AC , Woods M , Fu B , Yang F , Chen E , Ostermaier M , Stankovic T , Ponstingl H , et al. ATM orchestrates the DNA-damage response to counter toxic non-homologous end-joining at broken replication forks. Nat Commun. 2019; 10:87. 10.1038/s41467-018-07729-2. 30622252 PMC6325118

[9] Davis AJ , Chen DJ . DNA double strand break repair via non-homologous end-joining. Transl Cancer Res. 2013; 2:130–43. 10.3978/j.issn.2218-676X.2013.04.02. 24000320 PMC3758668

[10] Fousteri M , Mullenders LH . Transcription-coupled nucleotide excision repair in mammalian cells: molecular mechanisms and biological effects. Cell Res. 2008; 18:73–84. 10.1038/cr.2008.6. 18166977

[11] Robertson AB , Klungland A , Rognes T , Leiros I . DNA repair in mammalian cells: Base excision repair: the long and short of it. Cell Mol Life Sci. 2009; 66:981–93. 10.1007/s00018-009-8736-z. 19153658 PMC11131461

[12] Li GM . Mechanisms and functions of DNA mismatch repair. Cell Res. 2008; 18:85–98. 10.1038/cr.2007.115. 18157157

[13] Hill HA , Qi X , Jain P , Nomie K , Wang Y , Zhou S , Wang ML . Genetic mutations and features of mantle cell lymphoma: a systematic review and meta-analysis. Blood Adv. 2020; 4:2927–38. 10.1182/bloodadvances.2019001350. 32598477 PMC7362354

[14] Guarini A , Marinelli M , Tavolaro S , Bellacchio E , Magliozzi M , Chiaretti S , De Propris MS , Peragine N , Santangelo S , Paoloni F , Nanni M , Del Giudice I , Mauro FR , et al. ATM gene alterations in chronic lymphocytic leukemia patients induce a distinct gene expression profile and predict disease progression. Haematologica. 2012; 97:47–55. 10.3324/haematol.2011.049270. 21993670 PMC3248930

[15] Parvin S , Ramirez-Labrada A , Aumann S , Lu X , Weich N , Santiago G , Cortizas EM , Sharabi E , Zhang Y , Sanchez-Garcia I , Gentles AJ , Roberts E , Bilbao-Cortes D , et al. LMO2 Confers Synthetic Lethality to PARP Inhibition in DLBCL. Cancer Cell. 2019; 36:237–49.e6. 10.1016/j.ccell.2019.07.007. 31447348 PMC6752209

[16] Ralhan R , Kaur J . Alkylating agents and cancer therapy. Expert Opin Ther Pat. 2007; 17:1061–75. 10.1517/13543776.17.9.1061.

[17] Peng Y , Pei H . DNA alkylation lesion repair: outcomes and implications in cancer chemotherapy. J Zhejiang Univ Sci B. 2021; 22:47–62. 10.1631/jzus.B2000344. 33448187 PMC7818015

[18] Wang L , Li LR . R-CHOP resistance in diffuse large B-cell lymphoma: biological and molecular mechanisms. Chin Med J (Engl). 2020; 134:253–60. 10.1097/CM9.0000000000001294. 33323828 PMC7846449

[19] Lalic H , Aurer I , Batinic D , Visnjic D , Smoljo T , Babic A . Bendamustine: A review of pharmacology, clinical use and immunological effects (Review). Oncol Rep. 2022; 47:114. 10.3892/or.2022.8325. 35506458 PMC9100486

[20] Jaspers NG , Raams A , Kelner MJ , Ng JM , Yamashita YM , Takeda S , McMorris TC , Hoeijmakers JH . Anti-tumour compounds illudin S and Irofulven induce DNA lesions ignored by global repair and exclusively processed by transcription- and replication-coupled repair pathways. DNA Repair (Amst). 2002; 1:1027–38. 10.1016/s1568-7864(02)00166-0. 12531012

[21] Koeppel F , Poindessous V , Lazar V , Raymond E , Sarasin A , Larsen AK . Irofulven cytotoxicity depends on transcription-coupled nucleotide excision repair and is correlated with XPG expression in solid tumor cells. Clin Cancer Res. 2004; 10:5604–13. 10.1158/1078-0432.CCR-04-0442. 15328203

[22] McDermott J , Sturtevant D , Kathad U , Varma S , Zhou J , Kulkarni A , Biyani N , Schimke C , Reinhold WC , Elloumi F , Carr P , Pommier Y , Bhatia K . Artificial intelligence platform, RADR®, aids in the discovery of DNA damaging agent for the ultra-rare cancer Atypical Teratoid Rhabdoid Tumors. Frontiers Drug Discov. 2022; 2:1033395. 10.3389/fddsv.2022.1033395.

[23] Beeharry N , Rattner JB , Bellacosa A , Smith MR , Yen TJ . Dose dependent effects on cell cycle checkpoints and DNA repair by bendamustine. PLoS One. 2012; 7:e40342. 10.1371/journal.pone.0040342. 22768280 PMC3386996

[24] Sawant A , Kothandapani A , Zhitkovich A , Sobol RW , Patrick SM . Role of mismatch repair proteins in the processing of cisplatin interstrand cross-links. DNA Repair (Amst). 2015; 35:126–36. 10.1016/j.dnarep.2015.10.003. 26519826 PMC4651805

[25] Poindessous V , Koeppel F , Raymond E , Comisso M , Waters SJ , Larsen AK . Marked activity of irofulven toward human carcinoma cells: comparison with cisplatin and ecteinascidin. Clin Cancer Res. 2003; 9:2817–25. 12855662

[26] Barretina J , Caponigro G , Stransky N , Venkatesan K , Margolin AA , Kim S , Wilson CJ , Lehár J , Kryukov GV , Sonkin D , Reddy A , Liu M , Murray L , et al. The Cancer Cell Line Encyclopedia enables predictive modelling of anticancer drug sensitivity. Nature. 2012; 483:603–7. 10.1038/nature11003. 22460905 PMC3320027

[27] Vivian J , Rao AA , Nothaft FA , Ketchum C , Armstrong J , Novak A , Pfeil J , Narkizian J , Deran AD , Musselman-Brown A , Schmidt H , Amstutz P , Craft B , et al. Toil enables reproducible, open source, big biomedical data analyses. Nat Biotechnol. 2017; 35:314–16. 10.1038/nbt.3772. 28398314 PMC5546205

[28] Iorio F , Knijnenburg TA , Vis DJ , Bignell GR , Menden MP , Schubert M , Aben N , Gonçalves E , Barthorpe S , Lightfoot H , Cokelaer T , Greninger P , van Dyk E , et al. A Landscape of Pharmacogenomic Interactions in Cancer. Cell. 2016; 166:740–54. 10.1016/j.cell.2016.06.017. 27397505 PMC4967469

[29] Williamson CT , Muzik H , Turhan AG , Zamò A , O’Connor MJ , Bebb DG , Lees-Miller SP . ATM deficiency sensitizes mantle cell lymphoma cells to poly(ADP-ribose) polymerase-1 inhibitors. Mol Cancer Ther. 2010; 9:347–57. 10.1158/1535-7163.MCT-09-0872. 20124459 PMC3729269

[30] Rossi A , Orecchioni S , Falvo P , Tabanelli V , Baiardi E , Agostinelli C , Melle F , Motta G , Calleri A , Fiori S , Corsini C , Casadei B , Mazzara S , et al. Dual targeting of the DNA damage response pathway and BCL-2 in diffuse large B-cell lymphoma. Leukemia. 2022; 36:197–209. 10.1038/s41375-021-01347-6. 34304248 PMC8727301

[31] Munawar U , Roth M , Barrio S , Wajant H , Siegmund D , Bargou RC , Kortüm KM , Stühmer T . Assessment of TP53 lesions for p53 system functionality and drug resistance in multiple myeloma using an isogenic cell line model. Sci Rep. 2019; 9:18062. 10.1038/s41598-019-54407-4. 31792264 PMC6889167

[32] Chang BH , Johnson K , LaTocha D , Rowley JS , Bryant J , Burke R , Smith RL , Loriaux M , Müschen M , Mullighan C , Druker BJ , Tyner JW . YM155 potently kills acute lymphoblastic leukemia cells through activation of the DNA damage pathway. J Hematol Oncol. 2015; 8:39. 10.1186/s13045-015-0132-6. 25895498 PMC4408565

[33] Reinhold WC , Sunshine M , Liu H , Varma S , Kohn KW , Morris J , Doroshow J , Pommier Y . CellMiner: a web-based suite of genomic and pharmacologic tools to explore transcript and drug patterns in the NCI-60 cell line set. Cancer Res. 2012; 72:3499–511. 10.1158/0008-5472.CAN-12-1370. 22802077 PMC3399763

[34] Wang M , Han XH , Zhang L , Yang J , Qian JF , Shi YK , Kwak LW , Romaguera J , Yi Q . Bortezomib is synergistic with rituximab and cyclophosphamide in inducing apoptosis of mantle cell lymphoma cells *in vitro* and *in vivo* . Leukemia. 2008; 22:179–85. 10.1038/sj.leu.2404959. 17898787

[35] Wu W , Wang W , Franzen CA , Guo H , Lee J , Li Y , Sukhanova M , Sheng D , Venkataraman G , Ming M , Lu P , Gao A , Xia C , et al. Inhibition of B-cell receptor signaling disrupts cell adhesion in mantle cell lymphoma via RAC2. Blood Adv. 2021; 5:185–97. 10.1182/bloodadvances.2020001665. 33570628 PMC7805322

[36] Kordon MM , Zarębski M , Solarczyk K , Ma H , Pederson T , Dobrucki JW . STRIDE-a fluorescence method for direct, specific *in situ* detection of individual single- or double-strand DNA breaks in fixed cells. Nucleic Acids Res. 2020; 48:e14. 10.1093/nar/gkz1118. 31832687 PMC7026605

[37] Casimir L , Zimmer S , Racine-Brassard F , Jacques PÉ , Maréchal A . The mutational impact of Illudin S on human cells. DNA Repair (Amst). 2023; 122:103433. 10.1016/j.dnarep.2022.103433. 36566616

[38] Wang J , Wiltshire T , Wang Y , Mikell C , Burks J , Cunningham C , Van Laar ES , Waters SJ , Reed E , Wang W . ATM-dependent CHK2 activation induced by anticancer agent, irofulven. J Biol Chem. 2004; 279:39584–92. 10.1074/jbc.M400015200. 15269203

[39] Yang AL , Zdzienicka MZ , Simons JW , Waters R . The repair of 4-nitroquinoline-1-oxide induced DNA adducts in hypersensitive Chinese hamster mutants: lack of repair of UV induced (6-4) photoproduct correlates with reduced repair of adducts at the N2 of guanosine. Mutagenesis. 1991; 6:449–53. 10.1093/mutage/6.6.449. 1800891

[40] Thompson LH , Brookman KW , Dillehay LE , Carrano AV , Mazrimas JA , Mooney CL , Minkler JL . A CHO-cell strain having hypersensitivity to mutagens, a defect in DNA strand-break repair, and an extraordinary baseline frequency of sister-chromatid exchange. Mutat Res. 1982; 95:427–40. 10.1016/0027-5107(82)90276-7. 6889677

[41] Alekseev S , Ayadi M , Brino L , Egly JM , Larsen AK , Coin F . A small molecule screen identifies an inhibitor of DNA repair inducing the degradation of TFIIH and the chemosensitization of tumor cells to platinum. Chem Biol. 2014; 21:398–407. 10.1016/j.chembiol.2013.12.014. 24508195

[42] Szalat R , Samur MK , Fulciniti M , Lopez M , Nanjappa P , Cleynen A , Wen K , Kumar S , Perini T , Calkins AS , Reznichenko E , Chauhan D , Tai YT , et al. Nucleotide excision repair is a potential therapeutic target in multiple myeloma. Leukemia. 2018; 32:111–19. 10.1038/leu.2017.182. 28588253 PMC5720937

[43] Wang H , Zhang W , Yang J , Zhou K . The resistance mechanisms and treatment strategies of BTK inhibitors in B-cell lymphoma. Hematol Oncol. 2021; 39:605–15. 10.1002/hon.2933. 34651869 PMC9293416

[44] Skelding KA , Lincz LF . PARP Inhibitors and Haematological Malignancies-Friend or Foe? Cancers (Basel). 2021; 13:5328. 10.3390/cancers13215328. 34771492 PMC8582507

[45] Camacho E , Hernández L , Hernández S , Tort F , Bellosillo B , Beà S , Bosch F , Montserrat E , Cardesa A , Fernández PL , Campo E . ATM gene inactivation in mantle cell lymphoma mainly occurs by truncating mutations and missense mutations involving the phosphatidylinositol-3 kinase domain and is associated with increasing numbers of chromosomal imbalances. Blood. 2002; 99:238–44. 10.1182/blood.v99.1.238. 11756177

[46] Maifrede S , Martin K , Podszywalow-Bartnicka P , Sullivan-Reed K , Langer SK , Nejati R , Dasgupta Y , Hulse M , Gritsyuk D , Nieborowska-Skorska M , Lupey-Green LN , Zhao H , Piwocka K , et al. IGH/MYC Translocation Associates with BRCA2 Deficiency and Synthetic Lethality to PARP1 Inhibitors. Mol Cancer Res. 2017; 15:967–72. 10.1158/1541-7786.MCR-16-0468. 28634224 PMC5540764

