# Addendum: LP-284, a small molecule acylfulvene, exerts potent antitumor activity in preclinical non-Hodgkin’s lymphoma models and in cells deficient in DNA damage repair

**DOI:** 10.18632/oncotarget.28479

**Published:** 2023-07-20

**Authors:** Jianli Zhou, Drew Sturtevant, Cassie Love, Aditya Kulkarni, Neha Biyani, Umesh Kathad, Elizabeth Thacker, Sandeep Dave, Kishor Bhatia

**Affiliations:** ^1^Lantern Pharma Inc., Plano, TX 75024, USA; ^2^Department of Medicine, Duke University, Durham, NC 27708, USA; ^3^Data Driven Bioscience, Durham, NC 27707, USA

**This article has an addendum:** Wet-lab experiments conducted at Data Driven Bioscience, Rincon Biosciences, Reprocell Inc., intoDNA, and Toxys Europe were funded by Lantern Pharma Inc.

Original article: Oncotarget. 2023; 14:597–611. 597-611. https://doi.org/10.18632/oncotarget.28454

