# Supplementary material for: LP-284, a small molecule acylfulvene, exerts potent antitumor activity in preclinical non-Hodgkin's lymphoma models and in cells deficient in DNA damage repair
Source: Oncotarget. 2023 Jun 12;14:597–611. doi: 10.18632/oncotarget.28454 (PMC10259262; doi:10.18632/oncotarget.28454)
Supplement: Supplementary file 1 [file oncotarget-14-28454-s001.pdf]

LP-284, a small molecule acylfulvene, exerts potent antitumor activity in preclinical non-Hodgkin's lymphoma models and in cells deficient in DNA damage repair

SUPPLEMENTARY MATERIALS

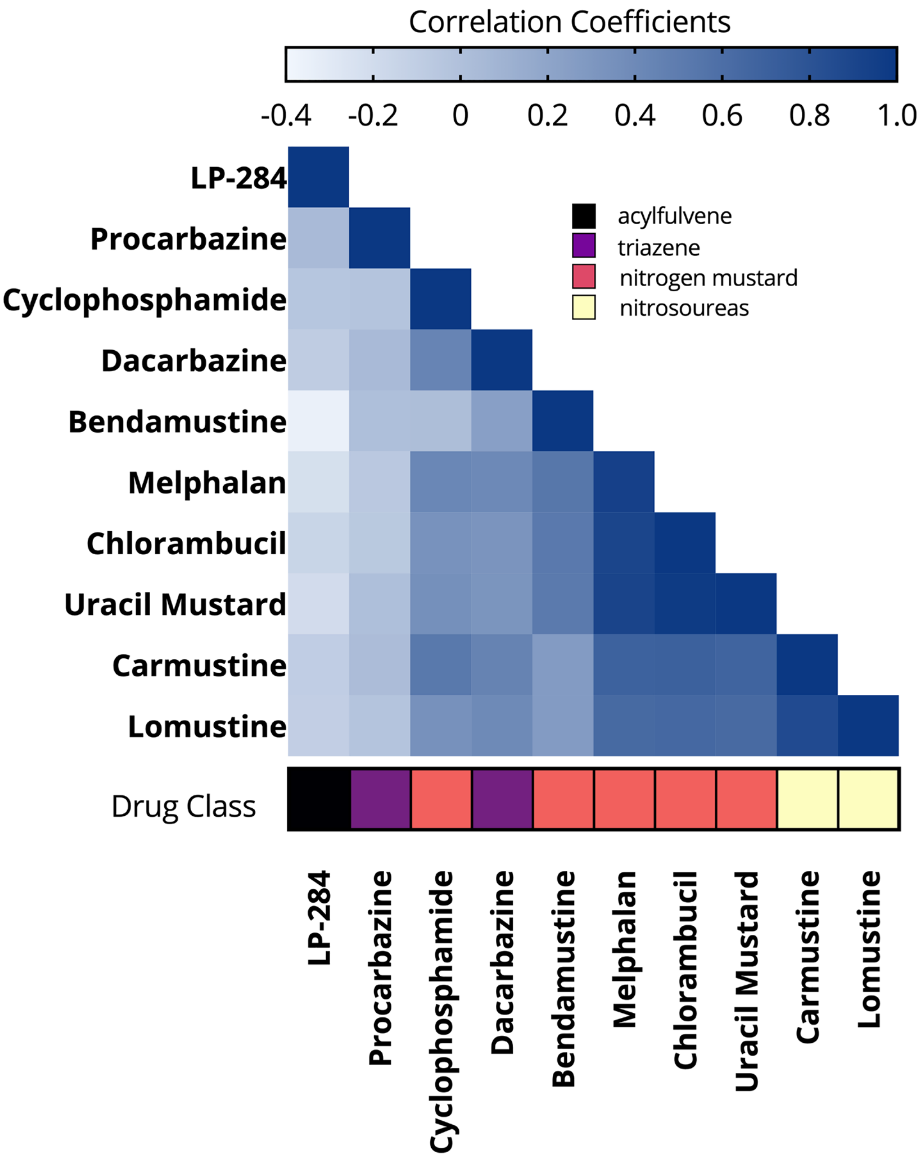

Supplementary Figure 1: LP-284 displays a distinct pattern of activities using the NCI drug correlation analysis. Pearson correlation analysis was conducted using IC50s of LP-284 and FDA-approved alkylating agents with indications involving hematologic malignancies. Correlation coefficients were retrieved from the CellminerCDB portal (https://discover.nci.nih.gov/rsconnect/cellminercdb/) and plotted in the heatmap.
